# Prevalence and patterns of drug resistance among pulmonary tuberculosis patients in Hangzhou, China

**DOI:** 10.1186/s13756-018-0348-7

**Published:** 2018-05-02

**Authors:** Qingchun Li, Gang Zhao, Limin Wu, Min Lu, Wei Liu, Yifei Wu, Le Wang, Ke Wang, Han-Zhu Qian, Li Xie

**Affiliations:** 1 Hangzhou Center for Disease Control and Prevention, Mingshi Road, Hangzhou City, 310021 Zhejiang Province China; 2 Department of Biostatistics, Yale School of Public Health, New Haven, Connecticut USA

**Keywords:** Tuberculosis, Drug sensitivity testing, Drug resistance, China

## Abstract

**Background:**

To evaluate prevalence and patterns of drug resistance among pulmonary tuberculosis (TB) patients in Hangzhou City, China.

**Methods:**

Sputum samples of smear positive TB patients enrolled in 2011 and 2015 were collected and tested for drug susceptibility, and demographic and medical record data were extracted from the electronic database of China Information System for Disease Control and Prevention. Chi-square test was used to compare drug resistance prevalence between new and treated patients and between male and female patients, and Chi-square test for trend was used to compare the prevalence over calendar years 2011 and 2015.

**Results:**

Of 1326 patients enrolled in 2015, 22.3% had resistance to any first-line anti-TB drugs and 8.0% had multi-drug resistance (MDR); drug resistance rates among previously treated cases were significantly higher than among new cases. Significant declines of resistance to isoniazid, rifampin, ethambutol and streptomycin, and MDR from 2011 to 2015 were observed among previously treated patients, while a significant decline of resistance to rifampin was observed among new cases.

**Conclusions:**

While the prevalence of acquired drug resistance decreased due to due to implementation of DOTS-Plus program, the prevalence of primary drug resistance due to transmission remained high. Greater efforts should be made to screen drug resistance for case finding and to reduce transmission through improving the treatment and management of drug-resistant patients.

## Background

China is one of the countries with the highest burden of tuberculosis (TB) disease in the world. Although its ranking in total TB cases dropped in 2015 from second to third behind India and Indonesia [1], the epidemic of drug-resistant TB (DR-TB) and multi-drug resistant TB (MDR-TB) is still a severe public health issue in China. A national survey published in 2012 showed 5.7% of new cases and 25.6% of previously treated cases had MDR-TB, both higher than the global averages [2]. The prevalence of DR-TB and MDR-TB varied geographically, and 57% TB patients were resistant to any first-line drugs and 24.1% were resistant to multiple drugs in high-burden regions [3, 4]. Studies have been conducted to investigate the prevalence of TB drug resistance across the country in recent years [3, 5], but few have evaluated the temporal trend. A study in Shanghai City in the middle of China’s east coast found the drug resistance rates increased significantly from 2000 to 2003, and then stabilized during 2004–2006 [6]. A study among TB patients in Hangzhou City in east China showed DR-TB and MDR-TB prevalence was 31.3% and 11.6%, respectively [7]. Little is known about the patterns of drug resistance and recent trend of the epidemic in Hangzhou City. This study reports drug resistance patterns and the epidemic trend from 2011 to 2015 in Hangzhou City in eastern China.

## Methods

### Study population

Hangzhou City is located in eastern China, about a hundred miles away from Shanghai. It comprises 13 districts, one county-level city, and two counties, and has 7.2 million local residents and over 2 million migrant populations.

All smear-positive pulmonary TB patients who lived in Hangzhou in years 2011 and 2015 were included in this study. The highest value was used for analysis if patients had multiple drug susceptibility testing (DST) results in the study years.

### Data collection and bacteriologic examinations

TB is a notifiable disease in China. Over 68,000 health facilities report notifiable diseases to the national, real-time, internet-based disease reporting system, known as the China Information System for Disease Control and Prevention (CIS-DCP). Hangzhou City Center for Disease Control and Prevention (CDC) is authorized for access to the sociodemographic information and medical records of TB patients who live in Hangzhou in this system.

TB cases were diagnosed following Chinese clinical guideline for TB diagnosis and treatment. Three sputum samples were collected from each participant at different time points (clinic visit, early morning, and night) prior to initiation of treatment, and were examined for acid-fast bacilli (AFB). Two specimens with the highest bacterial counts were used for culture. TB culture was performed as follows: First, decontaminating and digesting the sputum with equal volume of 4% sodium hydroxide for 15 min; Then, inoculating 0.1 ml specimen into the Lowenstein–Jensen medium, and culturing it in incubator at 37 °C; After that, observing the colony growth, which was confirmed by microscopic examination for AFB through Ziehl-Neelsen staining.

Species identification of mycobacteria was performed by conventional biochemical tests. Drug sensitivity test was performed using the proportion method on Löwenstein-Jensen medium, with the following concentrations: 0.2 micrograms per milliliter (μg/ml) for isoniazid, 2.0 μg/ml for ethambutol, 2.0 μg/ml for ofloxacin, 4.0 μg/ml for streptomycin, 30 μg/ml for kanamycin, and 40 μg/ml for rifampin. The critical growth proportion for drug resistance was 1% for all drugs. All drugs were obtained from Sigma Life Science Company (USA). The standard sensitive strain H37Rv was tested in each set of the tests and again within each set if the batch of medium was changed. The drug sensitivity test result or the H37Rv should be sensitive. All drug sensitivity tests in years 2011 and 2015 were performed by the same staff in the TB reference laboratory at Hangzhou CDC, a part of World Health Organization/International Union against Tuberculosis and Lung Disease Global Project on Anti-Tuberculosis Drug Resistance Surveillance.

### Statistical analysis

Statistical analysis was conducted with SPSS 12.0 software. Chi-square tests or Fisher’s exact tests were used comparing drug resistance rates between new and treated patients and between male and female patients. Chi-square tests for trend were used for comparing the difference of drug resistance rates from 2011 to 2015. *P* value < 0.05 was considered statistically significant.

## Results

### Demographic characteristics of TB patients

The general characteristics and drug resistance rates of 1184 participants in 2011 were reported elsewhere [7]; of these participants, 903 (76.3%) were new TB patients and 281 (23.7%) were previously treated patients. In 2015, a total of 1888 smear-positive pulmonary TB patients who lived in Hangzhou were diagnosed, of whom 1583 (83.8%) had positive sputum culture results and 1332 (70.6%) were positive for *M. tuberculosis*. Six patients were excluded due to lack of drug sensitivity test results for first-line anti-TB drugs; therefore, 1326 (70.2%) patients were included in the analysis.

Of 1326 patients, 961 (72.5%) were male and 365 (27.5%) were female; 1305 (98.42%) were Han Chinese and 21 (1.58%) were other ethnic minorities; age ranged from 12 to 94 years (mean 54); 289 (21.8%) were migrants; 1020 (76.9%) were new cases and 306 (23.1%) were previously treated cases; 874 (65.9%) had a drug sensitivity test result for ofloxacin and 875 (66.0%) for kanamycin.

### TB drug resistance patterns in 2015

In 2015, about 18% (184/1020) new TB patients were resistant to at least one first-line drug, while the prevalence of drug resistance among previously treated patients was double (36.6%, 112/306) (Table 1). The majority of drug resistance cases had resistance to a single drug, such as streptomycin, isoniazid, rifampin, ofloxacin, ethambutol and kanamycin. Eight percent of TB patients had multi-drug resistance (MDR), 3.8% among new patients and 22.2% among previously treated patients; the common combinations of MDR were isoniazid with rifampin or streptomycin. One new patient and two previously treated patients had extensive drug resistance (XDR) (Table 1).

**Table 1:** Drug resistance patterns among 1326 tuberculosis patients in Hangzhou, China, 2015

	Type of TB resistance	New cases (*N* = 1020)		Treated cases (*N* = 306)	
		n	%	n	%
Resistance to any first-line drugs		184	18.0	112	36.6
Resistance to individual drugs in any tests					
Isoniazid		102	10.0	89	29.1
Rifampin		57	5.6	78	25.5
Ethambutol		15	1.5	23	7.5
Streptomycin		119	11.7	58	19.0
Ofloxacin		20	2.0	15	4.9
Kanamycin		7	0.7	4	1.3
Resistance to single drug only					
Isoniazid		33	3.2	15	4.9
Rifampin		15	1.5	8	2.6
Ethambutol		2	0.2	0	0
Streptomycin		57	5.6	10	3.3
Ofloxacin		14	1.4	3	1.0
Kanamycin		2	0.2	0	0
Resistance to two drugs					
Isoniazid+ethambutol		1	0.1	1	0.3
Isoniazid+streptomycin		27	2.7	3	1.0
Rifampin+ethambutol		1	0.1	0	0
Rifampin+streptomycin		3	0.3	1	0.3
Ethambutol+streptomycin		1	0.1	1	0.3
Rifampin+ofloxacin		0	0	1	0.3
Streptomycin+ofloxacin		2	0.2	1	0.3
Isoniazid+ofloxacin		1	0.1	1	0.3
Streptomycin+ kanamycin		1	0.1	0	0
Isoniazid+rifampin		9	0.9	19	6.2
Resistance to three drugs					
Isoniazid+rifampin+ethambutol		0	0	6	2.0
Isoniazid+rifampin+Streptomycin		16	1.6	24	7.8
Ethambutol+streptomycin+ofloxacin		0	0	1	0.3
Isoniazid+rifampin+ofloxacin		1	0.1	1	0.3
Isoniazid+rifampin+kanamycin		1	0.1	0	0
Isoniazid+ethambutol+ofloxacin		0	0	1	0.3
Resistance to four drugs					
Isoniazid+rifampin+ethambutol+streptomycin		8	0.8	10	3.3
Isoniazid+rifampin+ethambutol+ofloxacin		0	0	2	0.7
Isoniazid+rifampin+streptomycin+ofloxacin		1	0.1	1	0.3
Isoniazid+rifampin+streptomycin+kanamycin		0	0	2	0.7
Resistance to five drugs					
Isoniazid+rifampin+ethambutol+streptomycin+ofloxacin		0	0	2	0.7
Isoniazid+rifampin+ethambutol+streptomycin+kanamycin		1	0.1	0	0
Multi-drug resistance (MDR)		38	3.7	68	22.2
Extensive drug resistance (XDR)					
Isoniazid+rifampin+ofloxacin+kanamycin (or cycloserine)^a^		1	0.1	2	0.7

^a^Of these 3XDR patients, two were resistant to kanamycin, and one to cycloserine

The difference in the prevalence of resistance to any single drug or to multiple drugs between new and treated patients was statistically significant (Table 2). This difference was same for both male and female patients, separately (not shown in tables). There was no statistically significant difference of drug resistance prevalence between male and female participants, and no difference by age group (not shown in tables).

**Table 2:** Comparison of drug resistance among 1326 new and treated tuberculosis cases in Hangzhou, China, 2015

Type of TB resistance	All (*N* = 1326) n, %	New cases (*N* = 1020) n, %	Treated cases (*N* = 306) n, %	χ^2^	*P*
Resistance to any first-line drugs	296 (22.3)	184 (18.0)	112 (36.6)	46.8	< 0.01
Isoniazid	191(14.40)	102 (10.00)	89 (29.08)	69.5	< 0.01
Rifampin	135 (10.2%)	57 (5.6)	78 (25.5)	102.0	< 0.01
Ethambutol	38 (2.9)	15 (1.5)	23 (7.5)	30.9	< 0.01
Streptomycin	177(13.4)	119 (11.7)	58 (19.0)	10.8	< 0.01
Multi-drug resistance (MDR)	106 (8.0)	38 (3.8)	68 (22.2)	109.5	< 0.01

### Time trend of drug resistance from 2011 to 2015

A significant decline of resistance to any first-line drugs from 2011 to 2015 was observed: from 31.3% to 22.3% among all TB patients, and from 23.4% to 18% in new and 57% to 36.6% in previously treated patients (*P* < 0.01). There were significant declines in resistance to isoniazid, rifampin, ethambutol, streptomycin, and multi-drugs among previously treated patients, while among new patients there was a significant decline for rifampin only (Table 3).

**Table 3:** Prevalence trend of drug resistance among tuberculosis patients in Hangzhou, China, from 2011 to 2015

Type of TB resistance	Treatment history	2011 (*N* = 1184)	2015 (*N* = 1326)	χ^2^	*P*
Resistance to any first-line drugs	All	371 (31.3)	296 (22.3)	26.0	< 0.01
	New cases	211 (23.4)	184 (18.0)	8.3	< 0.01
	Treated cases	160 (57.0)	112 (36.6)	24.4	< 0.01
Isoniazid	All	231 (19.5)	191 (14.4)	11.7	< 0.01
	New cases	103 (11.4)	102 (10.0)	1.0	0.32
	Treated cases	128 (45.6)	89 (29.1)	17.1	< 0.01
Rifampin	All	201 (17.0)	135 (10.2)	24.9	< 0.01
	New cases	82 (9.1)	57 (5.6)	8.7	< 0.01
	Treated cases	119 (42.4)	78 (25.5)	18.7	< 0.01
Ethambutol	All	60 (5.1)	38 (2.9)	8.1	< 0.01
	New cases	13 (1.4)	15 (1.5)	0.0	0.95
	Treated cases	47 (16.7)	23 (7.5)	11.8	< 0.01
Streptomycin	All	203 (17.2)	177 (13.4)	7.0	< 0.01
	New cases	110 (12.2)	119 (11.7)	0.1	0.73
	Treated cases	93 (33.1)	58 (19.0)	15.3	< 0.01
Multi-drug resistance (MDR)	All	137 (11.6)	106 (8.0)	9.2	< 0.01
	New cases	37 (4.1)	38 (3.7)	0.2	0.67
	Treated cases	100 (35.6)	68 (22.2)	12.8	< 0.01

## Discussion

Our study showed that 22.3% of TB patients in Hangzhou City in 2015 were resistant to at least one first-line anti-TB drugs and 8.0% were MDR, and the prevalence of MDR was lower among new cases (3.7%) than among treated cases (22.2%). The MDR prevalence is comparable to the global average, e.g., 3.3% among new cases and 20% among previously treated cases [1]. Among TB patients in Zhejiang province where Hangzhou City is located, 23.6% were resistant to any first-line drugs and 5.0% were MDR [8]. The prevalence rates of any first-line drug resistance and MDR in six Chinese provinces were 23.4% and 13.5%, respectively [9], whereas in other areas, the rate of resistance to any first-line drugs ranged from 16.6% and 57%, and MDR from 4.0% to 24.1% [5, 6, 10–13]. In summary, the drug resistance prevalence in Hangzhou City was in the lower range of the epidemics in China.

Studies have shown that there is increasing or persistently high prevalence of drug resistance among TB patients in Mainland China [3, 5, 6, 14], a review showed that primary MDR-TB prevalence in China was below 4.0% before 1995, and reached 10% by 2005; The acquired MDR-TB prevalence increased from 5% in 1995 to 32.4% in 1990 and then stayed around 30% until 2005 [15]. We observed significant decline of TB drug resistance in Hangzhou City, particularly among previous diagnosed TB patients, and the findings have significant implications. First, primary drug resistance among treatment-naïve TB patients is caused by transmission; while drug resistance among treated patients can also be acquired due to inappropriate treatment. The decline of resistance to most first-line drugs among treated patients, but only to rifampin among new patients during 2011–2015 suggests that the prevalence of acquired drug resistance (ADR) mutations in treated patients declined, but the prevalence of transmitted drug resistance (TDR) mutations remained high. This decline may be due to the improvement of TB treatment and management in Hangzhou City, as it has implemented the DOTS Plus program—a DOTS program with components for MDR TB diagnosis, management, and treatment. A recent study in Shanghai showed the primary resistance due to exogenous reinfection was the major cause of drug resistance among treated TB patients [16], and this observation was also confirmed in other parts of China [17]. Another study found that 60% MRD patients had primary drug resistance attributable to transmission [18]. It is suggested that more efforts are needed to enhance detection, treatment and management of drug resistant patients, and more effective strategies are needed to prevent and interrupt transmission of drug resistant tuberculosis.

Second, the widely used regimens for both new and treated TB patients in China are two months of isoniazid, rifampicin, pyrazinamide and ethambutol followed by four months of isoniazid and rifampicin (2HRZE/4HR) or two months of isoniazid, rifampicin, pyrazinamide, ethambutol and streptomycin followed by six months of isoniazid and rifampicin (2HRZES/6HR) [19].Previous studies showed that about 90% TB patients with resistance to rifampin were also resistant to isoniazid, so drug sensitivity test of rifampin could serve as an index for screening MDR [14]. In our study, 78% patients with resistance to rifampin were MDR. Rifampin resistance is associated with poorer clinical outcomes and requires an increase in duration of therapy. Although the drug resistance was relatively low among the new cases, 37% of MDR patients and 62% of patients with resistance to any first-line anti-TB drugs were from the new cases [20]. Therefore, newly diagnosed patients in economically developed areas should be screened for drug resistance prior to initiate TB treatment [20]. The findings are similar to those from a study in Taiwan, which showed that the acquired MDR-TB prevalence was significantly lower after the implementation of the DOTS and DOTS-plus programmes, while the primary MDR-TB prevalence remained stable [21]. The time trends of drug resistance prevalence varied geographically. A meta-analysis published in 2017 revealed that the MDR TB prevalence among newly diagnosed in Ethiopia in East Africa was 1.7% (95% confidence interval [CI], 1.2–2.3%) and among previously treated TB patients, 14.1% (95% CI, 10.9–17.2%); The overall MDR-TB prevalence showed a stable time trend over the past 10 years [22]. Another meta analysis of the studies conducted in India revealed a worsening trend in DR-TB between the two study decades (decade 1 from 1995 to 2005: 37.7% [95% CI, 29.0–46.4%] vs decade 2 from 2006 to 2015: 46.1% [95% CI, 39.0–53.2%]); The pooled estimate of MDR-TB resistance was higher in previously treated patients (decade 1: 29.8% [95% CI, 20.7–39.0%]; decade 2: 35.8% [95% CI, 29.2–42.4%]) as compared with the newly diagnosed cases (decade 1: 4.1% [95% CI, 2.7–5.6%]; decade 2: 5.6% [95% CI, 3.8–7.4%]) [23].

Our study has limitations. First, our study sample only included sputum smear-positive TB patients, but other study showed 17% of drug-resistant and 20% of MDR cases were linked to sputum smear-negative sources [24]. Therefore, the prevalence of drug resistance in our study may be overestimated, and our study findings may not be extrapolated to sputum smear-negative TB cases. Second, we did not do genotyping of TB infections, so we were unable to ascertain the sources of drug-resistant strains among previously treated cases. Third, we did not perform drug sensitivity testing for second-line anti-TB drugs for all MDR patients, the estimations of resistance to second-line anti-TB drugs and extensive drug resistance might be biased.

## Conclusions

In summary, our study found higher prevalence of drug resistance and MDR among treated TB patients than among new patients in Hangzhou City, and showed a decreasing trend from years 2011–2015. DOTS-Plus program should be expanded, and greater efforts should be made to screen drug resistance for case finding and to reduce transmission through improving the treatment and management of drug-resistant patients.

## Abbreviations

AFB: acid-fast bacilli
CDC: Center for Disease Control and Prevention
CIS-DCP: System for Disease Control and Prevention
DR-TB: drug-resistant TB
DST: drug susceptibility testing
MDR: multi-drug resistance
TB: tuberculosis
XDR: extensive drug resistance
